# Case report: A novel *WASHC5* variant altering mRNA splicing causes spastic paraplegia in a patient

**DOI:** 10.3389/fgene.2023.1205052

**Published:** 2023-10-31

**Authors:** Shan-Yu Gao, Yu-Xing Liu, Yi Dong, Liang-Liang Fan, Qi Ding, Lv Liu

**Affiliations:** ^1^ Department of Neurology, Changshu No. 2 People’s Hospital, Changshu, China; ^2^ Department of Cell Biology, School of Life Science, Central South University, Changsha, China; ^3^ Department of Pulmonary and Critical Care Medicine, Research Unit of Respiratory Disease, Hunan Diagnosis and Treatment Center of Respiratory Disease, The Second Xiangya Hospital, Central South University, Changsha, China; ^4^ Department of Nephrology, Xiangya Hospital Central South University, Changsha, China

**Keywords:** *WASHC5*, spastic paraplegia, splicing variant, heterozygote, whole-exome sequencing

## Abstract

**Background:** Hereditary spastic paraplegia (HSP) is a progressive upper-motor neurodegenerative disease. Mutations in the *WASHC5* gene are associated with autosomal dominant HSP, spastic paraplegia 8 (SPG8). However, due to the small number of reported cases, the exact mechanism remains unclear.

**Method:** We report a Chinese family with HSP. The proband was referred to our hospital due to restless leg syndrome and insomnia. The preliminary clinical diagnosis of the proband was spastic paraplegia. Whole-exome sequencing (WES) and RNA splicing analysis were conducted to evaluate the genetic cause of the disease in this family.

**Results:** A novel splice-altering variant (c.712–2A>G) in the *WASHC5* gene was detected and further verified by RNA splicing analysis and Sanger sequencing. Real-time qPCR analysis showed that the expression of genes involved in the Wiskott–Aldrich syndrome protein and SCAR homolog (WASH) complex and endosomal and lysosomal systems was altered due to this variant.

**Conclusion:** A novel heterozygous splice-altering variant (c.712–2A>G) in the *WASHC5* gene was detected in a Chinese family with HSP. Our study provided data for genetic counseling to this family and offered evidence that this splicing variant in the *WASHC5* gene is significant in causing HSP.

## Introduction

Hereditary spastic paraplegias (HSPs) are a group of genetically and clinically heterogeneous neurodegenerative diseases which involve the corticospinal tracts. Being a rare disease, the prevalence of HSP ranges from 2 to 5 in 100,000 cases worldwide (Shribman et al., 2019). Clinically, HSPs can be categorized as pure forms or complex forms. Cases with pure HSP form usually present with spasticity and weakness of the lower extremities but without other complications. Differently, apart from leg spasticity, cases with complex HSP forms usually have additional signs and symptoms, including ataxia, peripheral neuropathy, and intellectual disability and other clinical manifestations (de Bot et al., 2013; Murala et al., 2021).

In addition to the clinical phenotype, HSPs can also be categorized into different spastic paraplegia (SPG) types according to the inheritance pattern and pathophysiological molecular mechanism. As one of the most heterogeneous genetically neurodegenerative diseases, approximately 57 genetic loci (SPG1–57) and 37 causative genes have been reported to be associated with HSP and present with different hereditary patterns including autosomal dominant (AD) (SPG3A, SPG4, SPG6, SPG8–10, SPG12, SPG13, SPG17, SPG19, SPG29, SPG31, SPG33, SPG36–38, SPG41, SPG42, SPG72, and SPG73) and autosomal recessive (AR) (SPG5, SPG7, SPG11, SPG14, SPG15, SPG18, SPG20, SPG21, SPG23–28, SPG30, SPG32, SPG35, SPG39, SPG43–71, and SPG74) types (Marrone et al., 2022; Osmanovic et al., 2020; Prestsaeter et al., 2020; Rodrigues et al., 2020; Servelhere et al., 2021; Spagnoli et al., 2021; Yapijakis et al., 2021). Few other studies also reported some rare HSP families presenting with X-linked recessive (SPG1, SPG2, SPG16, SPG22, SPG34) or mitochondrial (MT-ATP6, MT-TI, MT-CO3, and MT-ND4) inheritance patterns (de Souza et al., 2017; Ishiura et al., 2014; Murala et al., 2021; Steyer et al., 2020). Among these SPG forms, SPG4 (OMIM#182601) is the most common form, followed by SPG3A (OMIM#182600), SPG8 (OMIM#603563) and SPG31 (OMIM#610250), which can be inherited in an AD manner (Ishiura et al., 2014). The remaining AD forms and most of the AR forms have only been reported in a few or single families (Meyyazhagan and Orlacchio, 2022). The enormous genetic heterogeneity of HSP brings great challenges to molecular diagnostics. In order to establish and refine the genotype–phenotype correlations, more HSP families are necessary to be identified and reported in the future.

Here, we report a Chinese family with HSP where the proband suffered from restless legs syndrome and insomnia. The preliminary clinical diagnosis of the proband was spastic paraplegia. Whole-exome sequencing (WES) was conducted to evaluate the genetic cause of HSP in this family. A novel splice variant in the *WASHC5* gene was identified and was confirmed to affect splicing. Thus, the proband was molecularly diagnosed as being affected by HSP type SPG8.

## Case presentation

### Clinical features

**FIGURE 1:**
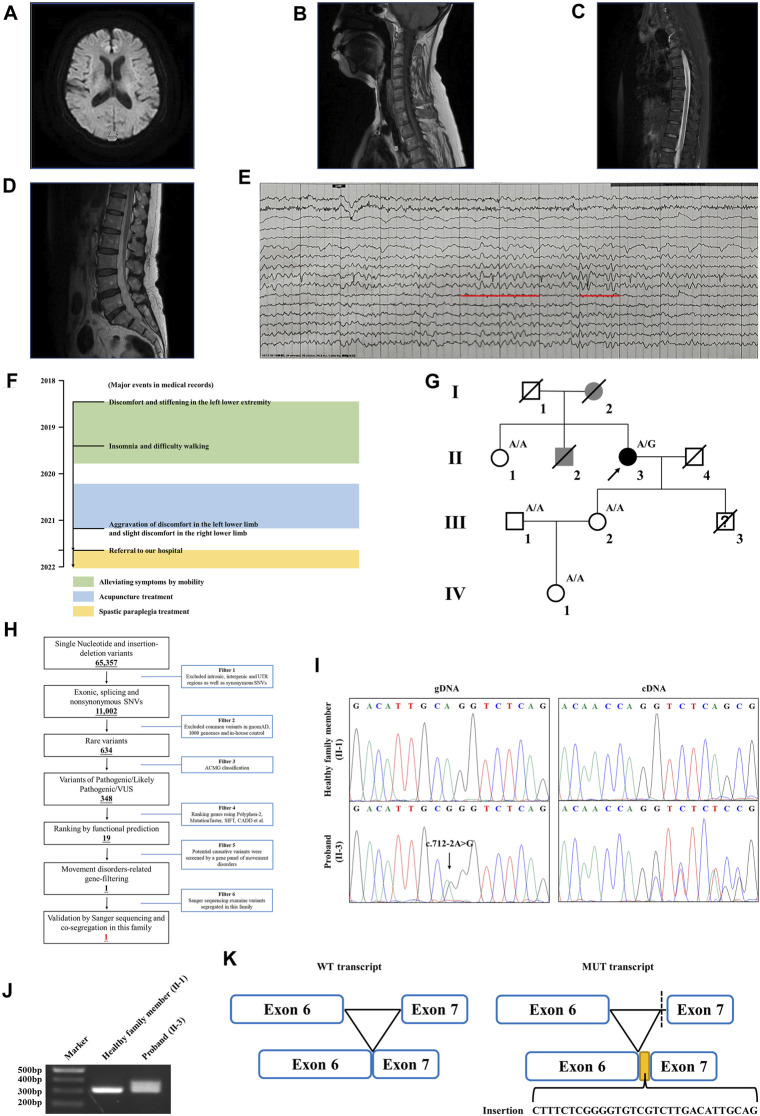
Clinical data of the family with HSP. **(A)** Brain MRI of the proband. The spinal cord MRI scans showed the **(B)** cervical spinal cord, **(C)** thoracic spinal cord, and **(D)** lumbar spinal cord of the proband. **(E)** The EEG of the proband. Red lines represent the 3–7 Hz medium–high amplitude θ rhythm. **(F)** Major events in the medical record of the proband. **(G)** Genealogy of this family having HSP. Squares and circles indicate male and female, respectively. Filled symbols indicate affected individuals. Black indicates an affected individual observed by clinical and molecular examination. Gray indicates an individual suspected of having spastic paraplegia, but that was not examined clinically and molecularly. Open symbols indicate unaffected individuals. Slashes indicate the individuals who are deceased. The arrow indicates the proband. “A/G” indicates the individuals who carried the heterozygous splice variant (c.712–2A>G) in the *WASHC5* gene. “A/A” indicates the individuals without the c.712–2A>G variant. **(H)** Schematic representation of the filtering strategies employed in the WES analysis. **(I)** Sanger sequencing showed the c.712–2A>G variant in the *WASHC5* gene (left) and the characterization of the anomalous splicing induced by the c.712–2A>G variant (right). **(J)** Agarose gel electrophoresis showed the PCR amplification of the *WASHC5* cDNA of the region from exon 6 to exon 7. The upper band corresponds to the mutant transcript. Lane 1: DNA size markers; lane 2: healthy family member (II-1) without the variant; and lane 3: the proband with the heterozygous c.712–2A>G variant. **(K)** Schematic representation of the anomalous mRNA splicing caused by the c.712–2A>G variant.

The proband is a 58-year-old woman from Jiangsu Province in China. She was referred to our hospital due to restless legs syndrome and insomnia. More than 2 years ago, the proband developed intolerable discomfort and stiffening in the left lower extremity without any trigger, which was temporarily relieved by mobility. A few months later, the symptoms were felt at rest or during sleep, which resulted in insomnia. There was patchy pain and numbness in the left sole which aggravated when the foot touched the floor, resulting in difficulty in walking. About a year ago, the proband started acupuncture treatment, but it did not improve her symptoms. In the past several months, the proband felt that her symptoms progressed. Discomfort in the left lower limb intensified, and concurrently, mild discomfort emerged in the right lower limb as well. The prolonged symptoms of discomfort and insomnia had contributed to the proband experiencing low mood and irritability. The proband is a non-smoker; had no previous history of hypertension, diabetes, cerebral infarction, or myocardial infarction; and denied bladder dysfunction. The proband’s cooperation during the examination was limited. She was alert and oriented, but in an anxious state. Her orientation was mostly intact, with no significant impairment in memory or calculation. Articulation was clear, bilateral nasolabial folds were symmetrical, and the tongue protruded centrally. Both pupils were equal and reactive to light, measuring 3.0 mm in diameter. Upon examination, it was found that the proband exhibited significant enhancement (++) in bilateral biceps and triceps reflexes. There was a slight elevation in the muscle tone in the left lower limb, with markedly increased bilateral patellar reflexes (+++), and a mild enhancement in bilateral ankle reflexes (+). The muscle strength of both limbs reached Grade 5, bilateral Babinski sign was negative, and bilateral sensory perception was symmetrical. The neck was relaxed, and there were no indications of Brudzinski’s sign or Kernig’s sign. No abnormalities were observed during the proband’s eye assessment. The patient exhibited normal hearing, and her intelligence was within the normal range. The proband’s cerebellar signs were within normal limits. The brain magnetic resonance imaging (MRI) of the proband showed mild brain atrophy (Figure 1A). The spinal cord MRI scans were normal (Figures 1B–D). The results of the laboratory examination are shown in the Supplementary Table S1. The results of the sensory nerve conduction velocity examination and motor nerve conduction velocity examination are shown in Supplementary Tables S2, S3, respectively. The findings indicated a reduction in the amplitude of the action potential of the left common peroneal nerve in the proband. The electromyography results revealed the absence of spontaneous muscle activity in the examined muscles of the proband’s left lower limb. However, due to inadequate patient cooperation during the testing procedure, other diagnostic data could not be obtained (Supplementary Table S4). There was no evidence indicating the presence of peripheral neuropathy in the proband. The electroencephalogram (EEG) showed mild abnormalities (Figure 1E). The self-rating anxiety scale (SAS) and self-rating depression scale (SDS) (Yue et al., 2020) were carried out, and the results showed mild anxiety (SAS score: 51, range 0–100) and severe depression (SDS score: 0.75, range 0–1). The major events as per medical records during 2018–2022 have been shown in Figure 1F. The proband was initially clinically diagnosed with spastic paraplegia.

Family history showed that the proband’s father (I-2) began experiencing difficulties with walking in his 50s, which gradually worsened over time, eventually necessitating the use of a wheelchair in his later years. The proband’s brother (II-2) had suffered from lower back pain and non-rheumatic joint pains during his lifetime. The deceased family members (I-2 and II-2) were not examined; however, they were considered affected according to relatives. Clinical information was not available for the proband’s younger son since he died in a car crash when he was 15. The proband’s sister (II-1) had hypertension but denied having walking problems. Other family members showed no obvious abnormalities (Figure 1G). As the proband’s father had similar walking problems, it was inferred that the disease was probably dominantly inherited.

### Genetic analysis

WES yielded 10.21 GB data with 98.1% coverage of the target regions and 96.0% of the target covered over ×10. All variants detected in the proband were initially screened and further filtered based on a gene panel of movement disorders, as outlined in Figure 1H; Supplementary Table S5. After analysis, a heterozygous splice variant (chr8:126088744T>C/NM_014846.4: c.712–2A>G) in *WASHC5* was identified in the proband (Figure 1I). The c.712–2A>G variant was not found in the 1,000 Genomes Project (1,000G; http://www.1000genomes.org/), the Exome Aggregation Consortium (ExAC) database (http://exac.broadinstitute.org), and the NHLBI Exome Sequencing Project Exome Variant Server (ESP6500) and has a frequency of 0.000004 (1/251100) in the Genome Aggregation Database (GnomAD; http://gnomad.broadinstitule.org). The c.712–2A>G variant is predicted to be pathogenic by MutationTaster (http://www.mutationtaster.org/). According to the American College of Medical Genetics and Genomics (ACMG) guidelines (Miller et al., 2021), with the following evidence: PVS1 and PM2, the c.712–2A>G variant is classified as “likely pathogenic.” Sanger sequencing was performed in all available family members. Co-segregation analysis showed that the c.712–2A>G variant was not present in the healthy members (II-1, III-1, III-2, and IV-1) in this family (Figure 1G). Consequently, the diagnosis of the proband was determined to be SPG8 type.

### RNA splicing analysis

To investigate the effect of the c.712–2A>G variant on pre-mRNA splicing, we further analyzed the *WASHC5* cDNA obtained from peripheral blood samples of the proband and family members. The WASHC5 reference sequence and coding region (NM_014846.4) were obtained from the National Center for Biotechnology Information (NCBI) database (https://www.ncbi.nlm.nih.gov/). Primers were designed to target *WASCH5* exons 5–7 to confirm aberrant splicing (forward: 5′-GCT​GGT​TTC​TTA​CTA​CCG​ATA​CA-3′; reverse: 5′-GGA​AGG​CTC​AAA​GTA​GAG​AAT​CA-3′). The DNA fragments were amplified by polymerase chain reaction (PCR). The PCR products were electrophoresed on agarose gels and were submitted for Sanger sequencing. Two transcripts were observed in gel electrophoresis. The Sanger sequence analysis of the region encompassing exon 6 and exon 7 found that the proband with the heterozygous c.712–2A>G variant showed two PCR products of 307 nucleotides and 337 nucleotides, corresponding to the wild-type and the mutated allele, respectively. The c.712–2A>G variant resulted in a 337 nucleotide aberrant PCR product in the proband. In contrast, relatives without the c.712–2A>G variant only had a single PCR product of the expected size (307 nucleotides) (Figure 1J). The Sanger sequencing of the mutant transcript confirmed that 30 nucleotides of intron 6 of the *WASHC5* are preserved in the open-reading frame. The retention of 30 nucleotides leads to the insertion of 10 aberrant amino acids (LSRGVVLTLR) at position 237 of the WASHC5 protein, thus resulting in an abnormally prolonged protein (1,169 aa) (Figures 1I, K).

### Bioinformatics analysis

**FIGURE 2:**
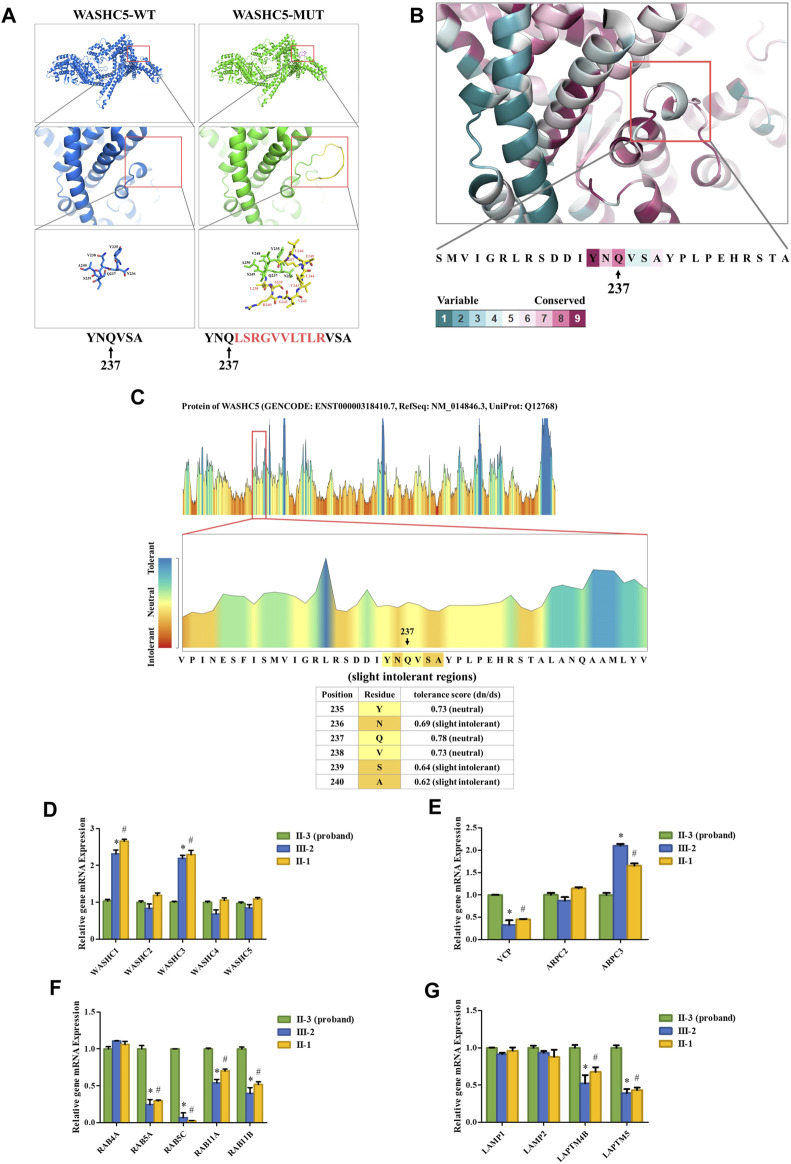
Bioinformatics analysis of the identified *WASHC5* splice-altering variant. **(A)** Structure prediction of the wildtype WASHC5 (WASHC5-WT) protein structure and the c.712–2A>G mutant WASHC5 (WASHC5-MUT) protein structure. **(B)** Conservation analysis of the related amino acid region was predicted by ConSurf Server software. **(C)** The affected amino acid region was predicted as “slight intolerant” by MetaDome server software. Real-time PCR analysis of the proband (II-3) and controls (II-1 and III-2). The relative mRNA expression levels of the genes encoding members of the WASH complex **(D)** VCP, ARPC2, and ARPC3 **(E)** in peripheral blood lymphocytes (PBLs) are depicted. mRNA abundance of endosomal-related genes **(F)** and lysosome-related genes **(G)** in PBL. The expression levels of each gene in the proband were set to “1”. The experiment was repeated more than three times. Data are presented as mean ± standard error of mean (SEM). Two groups were compared using the unpaired Student’s *t*-test. A *p*-value <0.05 was considered statistically significant. *p < 0.05, the healthy family member (III-2) vs. the proband; #*p* < 0.05, the healthy family member (II-1) vs. the proband.

SWISS-MODEL software (https://swissmodel.expasy.org/) showed that the splice variant may lead to an insertion of 10 amino acids behind Q237 in the mutated WASHC5 protein (Figure 2A). In addition, ConSurf Server software (http://consurf.tau.ac.il/) predicted that the 10 amino acids were inserted in the conserved region of the WASHC5 protein (Figure 2B). Furthermore, MetaDome software (https://stuart.radboudumc.nl/metadome/dashboard) indicated that the affected residues are located in the slightly intolerant region of the WASHC5 protein (Figure 2C).

### Real-time qPCR analysis

Strumpellin, encoded by the *WASHC5* gene, is a key member of the Wiskott–Aldrich syndrome protein and SCAR homolog (WASH) complex. Strumpellin can activate the ubiquitous actin-related protein 2/3 (ARP2/3) complex and interact with p97/VCP (Fokin et al., 2021; Song et al., 2018). Furthermore, the abnormal strumpellin may lead to defects in the endosomal and lysosomal systems (Gomez et al., 2012; Lee et al., 2020; Song et al., 2018). To investigate the functional involvement of the heterozygous splice-altering variant (NM_014846.4:c.712–2A>G) of *WASHC5* in this family, we next performed qPCR analysis. We isolated the mRNA from peripheral blood lymphocytes (PBLs) in the proband (II-3) and two healthy family members (II-1 and III-2). The proband (II-3) and the healthy family member (II-1) are siblings. All three subjects (II-1, II-3, and III-2) were female. The forward and reverse PCR primer sequences are listed in Supplementary Table S6. The relative mRNA levels of the target genes were normalized to that of *ACTB* and analyzed by the 2^−ΔΔCT^ method. We set the mRNA levels of each gene in the proband (II-3) as “1.” qPCR analysis indicated that the expression of WASH complex member-related genes in the proband, including *WASHC1* and *WASHC3*, was significantly decreased when compared with the healthy family members (Figure 2D). Compared with the healthy family members, the mRNA level of the *VCP* gene was significantly increased in the proband. We analyzed the expression of the *ARPC2* and *ARPC3* genes as representatives of the ARP2/3 complex. The results showed that there was a significant change in the proband’s *ARPC3* gene expression (Figure 2E). In addition, the transcriptional analysis revealed that the expression of endosomal-related genes (*RAB5A*, *RAB5C*, *RAB11A*, and *RAB11B*) and lysosome-related genes (*LAPTM4B* and *LAPTM5*) was significantly upregulated in the proband when compared to the healthy family members (Figures 2F, G).

## Discussion

In the current research, we described a Chinese family with HSP. The proband suffered from restless leg syndrome and insomnia. The preliminary clinical diagnosis of the proband was spastic paraplegia. WES was conducted to evaluate the genetic cause of disease in this family. A heterozygous splice-altering variant (c.712–2A>G) in the *WASHC5* gene was identified. Co-segregation analysis confirmed that this heterozygous splice variant was not present in the remaining unaffected family members (II-1, III-1, III-2, and IV-1). Two deceased family members (I-2 and II-2) were considered to be affected according to their relatives; however, they were deceased and could not be tested since their DNA was not available. Therefore, it was not possible to verify whether they carried the variant. Moreover, genetic analysis information was not available for the proband’s younger son (III-3) since he died in a car crash when he was 15. According to the literature, most of the symptoms of HSP are first observed in individuals aged 35–53, and intrafamilial phenotypic heterogeneity often existed (Valdmanis et al., 2007). Thus, although no walking problems were noticed before he died, we are not sure whether the proband’s son (III-3) was affected or not as he died before the age of onset. Based on the results of the co-segregation analysis, we have provided written genetic counseling to this family. It is reassuring to note that the proband’s sister (II-1) does not carry the c.712–2A>G variant, alleviating the need for undue concern in her case. Furthermore, we have determined that the proband’s offspring have not inherited the c.712–2A>G variant, indicating that its genetic impact can be excluded in future prenatal diagnostics.

Mutations in the *WASHC5* gene, also known as the *KIAA0196* gene, can lead to SPG8 (OMIM#603563). Since the splice-altering variant c.712–2A>G in the *WASHC5* gene was identified, the diagnosis of the proband was further determined as SPG8. SPG8 is a rare autosomal dominant HSP characterized by onset of progressive lower limb spasticity and hyperreflexia in adults, resulting in impaired gait and difficulty in walking. The age distribution of symptom onset is wide, and the severity is variable. Some patients may become wheelchair-bound after several decades. Other patients’ presentation of symptoms may be late in their mid-50s with very slow deterioration. Other features may include upper limb spasticity, impaired vibration sense in the distal lower limbs, and urinary urgency or incontinence (de Bot et al., 2013). Ichinose et al. (2016) described the clinical and genetic findings in a patient with SPG8. The patient suffered from insomnia due to the pain caused by the nocturnal muscle spasm of his legs. Brain MRI and spinal cord MRI showed no significant abnormal findings. Jahic et al. (2014) detected a novel strumpellin alteration in a family with clinically pure HSP. The proband reported in the research of Jahic et al. (2014) had a 5-year history of progressively worsening ability to walk and complained of restless legs. Similarly, the proband in our study was referred to our hospital due to restless legs syndrome and insomnia. She had intolerable discomfort and spasticity in the left lower extremity. The symptoms were obvious at rest or during sleep, which resulted in difficulty sleeping. Moreover, the patients reported by de Bot et al. (2013) suffered from non-specific lower back pain and non-rheumatic joint pains, and this was the reason why the proband’s deceased brother in our study was considered to be affected. Notably, our proband showed asymmetric spasticity of a single lower extremity. To our knowledge, this phenotype has so far not been described in SPG8 patients. Thus, our proband’s phenotype expands the phenotypic spectrum of SPG8. Based on the genetic findings, the proband was managed with mecobalamin (1.5 mg/day), lorazepam (2 mg/day), and baclofen (30 mg/day, orally). Furthermore, a follow-up visit has been scheduled to ensure the patients benefit from personalized treatment.

**FIGURE 3:**
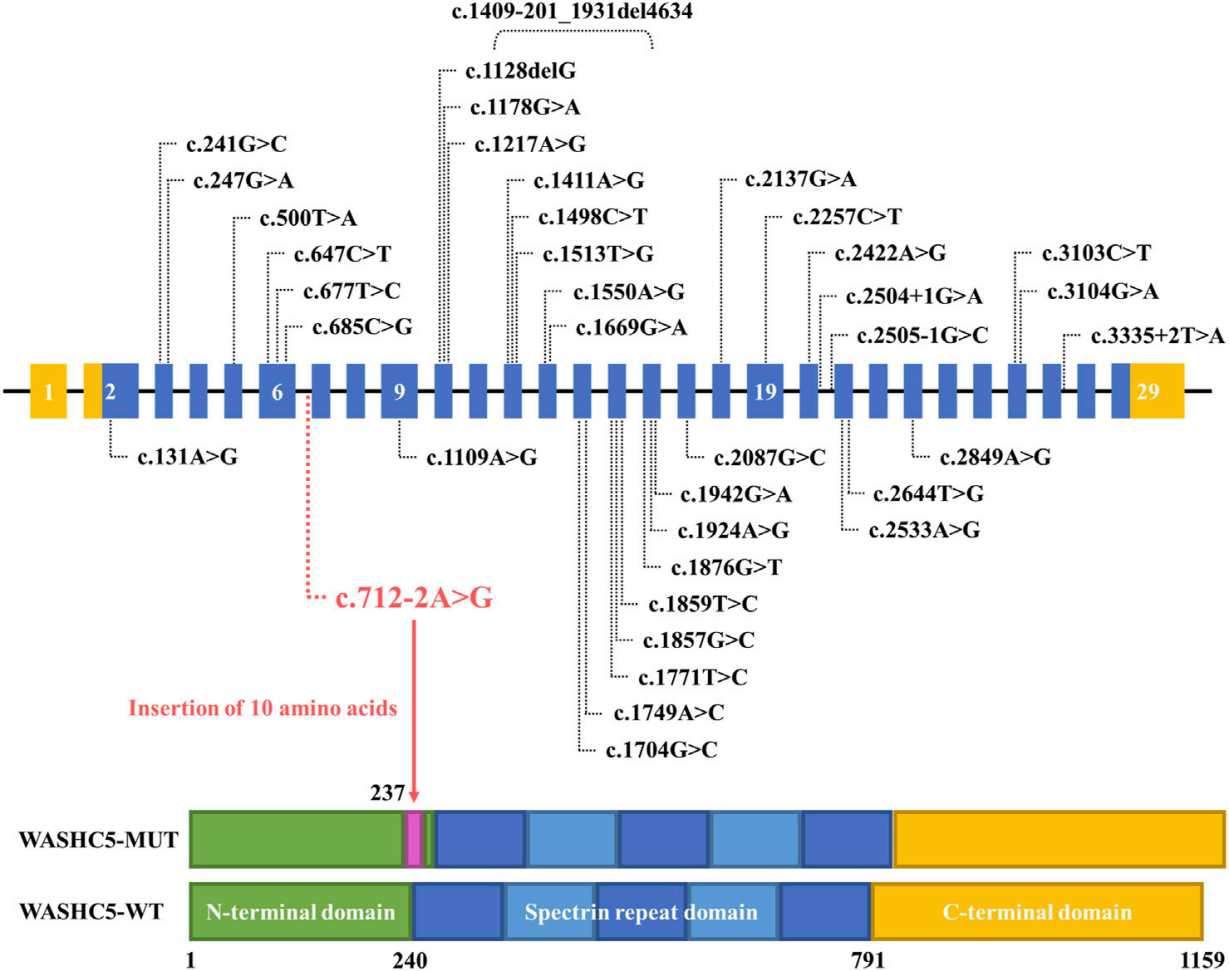
Schematic representation of all currently reported *WASHC5* mutations. The *WASHC5* gene is shown. The novel variant (c.712–2A>G) is marked in red letters.

Although approximately 57 genetic loci and 37 causative genes have been reported to be associated with HSPs (de Souza et al., 2017; Ishiura et al., 2014; Murala et al., 2021), only a few mutations have been reported as a cause of SPG8. To date, approximately 37 variants in *WASHC5*, including three splice mutations and one gross deletion variant, have been identified in a range of phenotypes (Elliott et al., 2013; Mulroy et al., 2021; D'Amore et al., 2018; Ma et al., 2018). Among them, only 24 mutations were reported in SPG8-affected individuals (Figure 3; Supplementary File S1). Here, we report the fourth splice-altering variant in the *WASHC5* gene in a HSP patient. RNA splicing analysis revealed that the c.712–2A>G variant gave rise to a 337-nucleotide aberrant transcript in the proband, compared with the expected size transcript (307 nucleotides) in the healthy control (Figure 1H). In addition, Sanger sequencing of the mutant transcript confirmed the retention of 30 nucleotides of intron 6 of *WASHC5* (Figures 1G, I). Furthermore, bioinformatics analysis predicted that the mutant transcript leads to the insertion of 10 aberrant amino acids in the intolerant region of the WASHC5 protein, breaking the original α-helix structure and resulting in an abnormally prolonged protein (Figures 2A, C). Given the degree of protein structure alteration, it was highly considered that the c.712–2A>G variant was responsible for the SPG8 in the proband. Performing additional functional analyses on the splice variant, such as investigating whether this variant affects protein stability and half-life, may yield further insights into the pathogenic mechanisms. Although the molecular mechanisms involved still require further exploration, our findings offer more evidence that splice variants of the *WASHC5* gene are significant in HSP. Notably, the c.712–2A>G variant reported in this study has not been published; therefore, it is considered novel.

The *WASHC5* gene encodes the strumpellin protein which is involved in the formation of the WASH complex. The WASH complex is a large five-protein assembly that consists of the WASH protein (encoded by the *WASHC1* gene), the FAM21 protein (encoded by the *WASHC2* gene), the coiled coil domain containing 53 protein (CCDC53) (encoded by the *WASHC3* gene), the strumpellin and WASH-interacting protein (SWIP) (encoded by the *WASHC4* gene), and the strumpellin protein (Assoum et al., 2020; Clemen et al., 2022; Song et al., 2018). Previous studies have reported that disruption of either one of the five WASH complex subunits makes the whole complex unstable and may disturb the quantity of other WASH complex members (Jahic et al., 2015; Song et al., 2018). Consistent with the previous study, the mRNA level of *WASHC5* in the proband was not altered by the c.712–2A>G variant. Interestingly, qPCR results revealed that the proband with the splice variant had lower mRNA levels of *WASHC1* and *WASHC3*. The expression of genes encoding other WASH complex members hardly changed (Figure 2D). The WASH complex is a nucleation promoting factor that activates the ARP2/3 complex and participates in the spatial and temporal control of actin assembly at endosomes (Song et al., 2018). WASH complex-mediated actin is essential for both endosomal and lysosomal networks (Courtland et al., 2021; Gomez et al., 2012). In this process, the strumpellin protein acts at the interface between actin regulation and endosomal membrane dynamics. Pathogenic *WASHC5* mutations in strumpellin can induce defects in endosomal fission or lysosome morphology, which further leads to HSP (Lee et al., 2020; Song et al., 2018). In our research, we found that the proband with the splice variant had a higher mRNA level of the *VCP* gene and lower mRNA level of the *ARPC3* gene. Furthermore, the expression of endosomal-related genes (*RAB5A*, *RAB5C*, *RAB11A*, and *RAB11B*) and lysosome-related genes (*LAPTM4B* and *LAPTM5*) changed dramatically in the proband when compared with the healthy family members. No changes in *RAB4A*, *LAMP1*, and *LAMP2* mRNA levels were detected (Figures 2F, G). These results indicate that both endosomal and lysosomal systems of the proband could be affected by the c.712–2A>G variant. This hypothesis will only be proven by further study. Additional functional analysis of the splice variant is recommended and may provide more information about the pathogenetic mechanism of HSP.

## Conclusion

We used WES to evaluate the genetic cause of the disease in a Chinese family with HSP. A novel heterozygous splice variant (c.712–2A>G) of the *WASHC5* gene was detected. The proband was further molecularly diagnosed with SPG8. Our study provided information for the genetic counseling of this family and offered more evidence that the splice variant of the *WASHC5* gene is significant cause of HSP.

## Data Availability

The datasets for this article are not publicly available due to concerns regarding participant/patient anonymity. Requests to access the datasets should be directed to the corresponding authors.
