# Supplementary material for: Case report: A novel WASHC5 variant altering mRNA splicing causes spastic paraplegia in a patient
Source: Front Genet. 2023 Oct 31;14:1205052. doi: 10.3389/fgene.2023.1205052 (PMC10644772; doi:10.3389/fgene.2023.1205052)
Supplement: Supplementary file 1 [file Table1.DOCX]

Supplementary Table 1. Results of serum and urine laboratory investigations in various measurements in the proband.

| Laboratory values | Observed values | Normal values (range) |
| --- | --- | --- |
| CRP (mg/L) | 0.6 | 0-5 |
| RBC (/L) | 3.54🞨10^12^ | 3.8-5.1🞨10^12^ |
| Hb (g/L) | 99 | 110-150 |
| PLT (/L) | 74🞨10^9^ | 125-350🞨10^9^ |
| WBC (/L) | 2.8🞨10^9^ | 3.5-9.5🞨10^9^ |
| Neutrophils (%) | 76.2 | 40-75 |
| ALC (/L) | 0.6🞨10^9^ | 1.1-3.2🞨10^9^ |
| EOSC (/L) | 0.01🞨10^9^ | 0.02-0.52🞨10^9^ |
| HCT (%) | 31.7 | 35-45 |
| MPV (fl) | 16.3 | 7-11 |
| P-LCR (%) | 67.8 | 15-60 |
| Ferritin (ng/ml) | 255.0 | 5-130 |
| P-RET (%) | 1.98 | 0.5-1.5 |
| ALT (U/L) | 8 | 5-35 |
| AST (U/L) | 11 | 8-40 |
| ALB (g/L) | 42.6 | 40-55 |
| Urea (mmol/L) | 5.3 | 2.8-7.2 |
| Creatinine (μmol/L) | 59 | 44-97 |
| Uric acid (μmol/L) | 296 | 155-357 |
| Serum potassium (mmol/L) | 4.10 | 3.5-5.5 |
| Serum sodium (mmol/L) | 142.2 | 135-145 |
| Serum calcium (mmol/L) | 2.22 | 2.25-2.75 |
| TG (mmol/L) | 0.62 | ＜1.7 |
| TC (mmol/L) | 3.85 | ＜5.18 |
| LDL-C (mmol/L) | 2.11 | ＜3.4 |
| HCY (μmol/L) | 9.5 | 5.08-15.39 |
| Folic acid (ng/ml) | 8.49 | 13.5-47.5 |
| Vitamin B12 (pg/ml) | 418.0 | 148-738 |
| HBALC (%) | 4.5 | 4-6 |
| FT3 (pmol/L) | 4.05 | 3.5-6.5 |
| FT4 (pmol/L) | 10.9 | 11.5-22.7 |
| TSH (uIU/ml) | 1.80 | 0.35-5.5 |

Abbreviations: ALB, albumin; ALC, absolute lymphocyte count; ALT, alanine aminotransferase; AST, aspartate amino transferase; CRP, C-reactive protein; EOSC, eosinophils count; FT3, free triiodothyronine; FT4, free thyroxine; HBALC, glycated hemoglobin; Hb, haemoglobin; HCT, hematocrit; HCY, homocysteine; LDLR, low density lipoprotein cholesterol; MPV, mean platelet volume; P-LCR, percentage of large platelets; P-RET, percentage of reticulocytes; PLT, platelet count; RBC, red blood cell count; TC, total cholesterol TG, triglyceride; TSH, thyroid stimulating hormone; WBC, white blood cell count.

Supplementary Table 2. Results of sensory nerve conduction velocity test in the proband.

| Nerve/body part | Latency  (ms) | Amplitude  (μV) | Conduction velocity  (m/s) | Distance  (mm) |
| --- | --- | --- | --- | --- |
| L-Peroneal nerve | | | | |
| Peroneal nerve | 2.03 | 27.9 (＞10) | 44 (42.3-53.3) | 90 |
| L-Superficial peroneal nerve | | | | |
| Lower leg | 2.03 | 15.8 (1-10) | 44 (40-68) | 90 |
| L- Medial plantar nerve | | | | |
| Toe (1) | 3.02 | 11.0 (≥1.6) | 43 (Average: 43.9) | 130 |

Abbreviations: L, left. Normal ranges are indicated in parentheses.

Supplementary Table 3. Results of motor nerve conduction velocity test in the proband.

| Nerve/body part | Latency  (ms) | Amplitude  (μV) | Conduction velocity  (m/s) | Distance  (mm) |
| --- | --- | --- | --- | --- |
| L-Common peroneal nerve | | | | |
| Ankle | 4.90 (3.4-5.0) | 1.5 (3.0-30.0) |  | 40 |
| Fibular head | 11.30 (3.4-5.0) | 1.5 (3.0-21.0) | 41 (43-57) | 260 |
| Popliteal fossa | 13.75 (3.4-5.0) | 1.5 (3.0-21.0) | 41 (44-57) | 100 |
| R-Common peroneal nerve | | | | |
| Ankle | 4.79 (3.4-5.0) | 2.1 (3.0-30.0) |  | 40 |
| Fibular head | 11.25 (3.4-5.0) | 1.8 (3.0-21.0) | 42 (43-57) | 270 |
| Popliteal fossa | 13.70 (3.4-5.0) | 1.7 (3.0-21.0) | 41 (44-57) | 100 |
| R-Common peroneal nerve (anterior tibial muscle recording) | | | | |
| Fibular head | 3.59 (3.4-5.0) | 4.8 (3.0-21.0) |  | 100 |
| Popliteal fossa | 5.57 (3.4-5.0) | 4.5 (3.0-21.0) | 51 (44-57) | 100 |
| L-Common peroneal nerve (anterior tibial muscle recording) | | | | |
| Fibular head | 3.91 (3.4-5.0) | 4.7 (3.0-21.0) |  | 100 |
| Popliteal fossa | 5.89 (3.4-5.0) | 4.9 (3.0-21.0) | 51 (44-57) | 100 |
| L-Tibial nerve | | | | |
| Ankle | 3.44 (2.96-4.96) | 12.8 (3.9-7.7) |  | 50 |
| Popliteal fossa | 11.82 (10.52-13.58) | 9.4 (2.9-7.3) | 42 (44.9-52.1) | 350 |

Abbreviations: L, left; R, right. Normal ranges are indicated in parentheses.

Supplementary Table 4. Results of electromyography in the proband.

| Examined muscle | Insertion potential | Relaxation | | | |  | Mild contraction | |  | Strong contraction | |
| --- | --- | --- | --- | --- | --- | --- | --- | --- | --- | --- | --- |
|  |  | Fibrillation | Positive sharp wave | Fasciculation | Myotonia |  | Latency | Polyphasic potentials |  | Waveform | Peak voltage |
| L-Tibialis anterior muscle | NA | - | - | NA | NA |  | NA | NA |  | NA | NA |
| L-Medial head of gastrocnemius | NA | - | - | NA | NA |  | NA | NA |  | NA | NA |
| L-Vastus medialis muscle | NA | - | - | NA | NA |  | NA | NA |  | NA | NA |

Abbreviations: “-”, negative; L, left; NA, not available.

Supplementary Table 5. The gene panel of movement disorders.

| *ABCB7* | *ABCD1* | *ABHD12* | *ACTB* | *ADCK3* | *AFG3L2* | *ALDH3A2* | *ANO10* |
| --- | --- | --- | --- | --- | --- | --- | --- |
| *ANO3* | *AP4B1* | *AP4E1* | *AP4M1* | *AP4S1* | *AP5Z1* | *APTX* | *ARSA* |
| *ARX* | *ASPA* | *ATCAY* | *ATL1* | *ATM* | *ATP13A2* | *ATP1A3* | *ATP2B3* |
| *ATP7B* | *B4GALNT1* | *BCKDHA* | *BCKDHB* | *BSCL2* | *C10orf2* | *C19ORF12* | *CA8* |
| *CACNA1A* | *CACNB4* | *CCT5* | *CIZ1* | *COQ2* | *COQ9* | *CP* | *CSTB* |
| *CYP27A1* | *CYP2U1* | *CYP7B1* | *DBT* | *DCAF17* | *DCTN1* | *DDC* | *DDHD1* |
| *DDHD2* | *DLAT* | *DLD* | *DNMT1* | *EIF2B1* | *EIF2B2* | *EIF2B3* | *EIF2B4* |
| *EIF2B5* | *EIF4G1* | *ERLIN2* | *FA2H* | *FBXO7* | *FGF14* | *FLVCR1* | *FTL* |
| *GALC* | *GAN* | *GBA* | *GBA2* | *GCDH* | *GCH1* | *GFAP* | *GJC2* |
| *GLB1* | *GNAL* | *GOSR2* | *GPR56* | *GRM1* | *HEXB* | *HPRT1* | *HSPD1* |
| *ITPR1* | *KCNA1* | *KCNC3* | *KCNJ10* | *KIAA0196* | *KIF1A* | *KIF1C* | *KIF5A* |
| *L1CAM* | *MARS2* | *MECP2* | *MMADHC* | *MRE11A* | *MTHFR* | *MTPAP* | *NIPA1* |
| *NKX2-1* | *NOL3* | *NPC1* | *NPC2* | *NUP62* | *OPA1* | *PANK2* | *PAX6* |
| *PDE8B* | *PDHA1* | *PDSS1* | *PDSS2* | *PDYN* | *PEX10* | *PEX7* | *PHYH* |
| *PIK3R5* | *PLA2G6* | *PLP1* | *PMM2* | *PNKD* | *PNPLA6* | *POLG* | *PRKCG* |
| *PRKRA* | *PRRT2* | *PSEN1* | *REEP1* | *RNASEH2A* | *RNASEH2B* | *RNASEH2C* | *RNF170* |
| *RTN2* | *SACS* | *SAMHD1* | *SERAC1* | *SETX* | *SGCE* | *SIL1* | *SLC16A2* |
| *SLC19A3* | *SLC1A3* | *SLC25A15* | *SLC2A1* | *SLC30A10* | *SLC33A1* | *SLC52A2* | *SLC6A3* |
| *SMPD1* | *SNCA* | *SPAST* | *SPG11* | *SPG20* | *SPG21* | *SPG7* | *SPR* |
| *SPTBN2* | *STUB1* | *SUOX* | *SYNE1* | *TAF1* | *TDP1* | *TECPR2* | *TGM6* |
| *TH* | *THAP1* | *TIMM8A* | *TMEM67* | *TOR1A* | *TREX1* | *TTBK2* | *TTC19* |
| *TTPA* | *TUBB4A* | *VAMP1* | *VCP* | *VLDLR* | *VPS13A* | *VPS37A* | *WDR45* |
| *WDR81* | *WWOX* | *ZFYVE26* | *ZFYVE27* | *ZNF592* |  |  |  |

Supplementary Table 6. Primers used in this study

| Gene | Forward primer | Reverse primer | Species |
| --- | --- | --- | --- |
| *WASHC1* | CTATGCCGTGCCCTTCATC | TGGGAGATCCTGCTGAAGAT | Human |
| *WASHC2* | ACGAAGAAGGAGATCTGTTCAA | TTTGAGTCTTTGTTTCTGATGAGG | Human |
| *WASHC3* | GGCAGACCTTTCACTTCGTATC | GCTTCAGGATGTGCTCCATT | Human |
| *WASHC4* | GTGGAAGGTGATTGCCAAATTC | GGACTACGTTCATCACCACTTC | Human |
| *WASHC5* | TTTCCAGAGAGTGCCTATCAAC | ATGCTCCGGCAAAGGATAC | Human |
| *VCP* | CCAGCCCAAGATGGATGAAT | CATCAGAAAGGACGATGCAAAC | Human |
| *ARPC2* | GGGTCCTCTATCATATTTCAAATCCTA | CCCGTACACCCTCTTTAATAACTC | Human |
| *ARPC3* | AGATGAAGTGATGAGAGCCTATTT | CTGGGTTTATCATTCTGAGGGT | Human |
| *ARPC4* | GTTGTGGAACGACACAACAAG | GCTGATGGTCACAGGTTGTA | Human |
| *RAB4A* | AGCAGGACAAGAACGATTCAG | TAGGTTTCTCGGCTGGTGATA | Human |
| *RAB5A* | AGAGGCAAGCAAGTCCTAAC | GACTGTGCTTCCTGGAAATCTA | Human |
| *RAB5C* | CTGCCTCTTCTTTCTCTCCATC | TGCTATGCAAAGAGGCACTTA | Human |
| *RAB11A* | AAGGCACAGATATGGGACAC | CACCTACAGCTCCACGATAAT | Human |
| *RAB11B* | TCAGCCTTGGATTCCACTAAC | GGTCTGCGATCTGTTTCTGT | Human |
| *LAMP1* | GGGACCGCGTGCATAAT | ATCTGATGGCAGGTCAAAGG | Human |
| *LAMP2* | AACCAGAAGCTGGAACCTATTC | TGAAGCAACCTTATCCTGAGTG | Human |
| *LAPTM4B* | TATTGAGTGCCCTGGCTGAT | CAATGGCAATGCACATGTTGG | Human |
| *LAPTM5* | CCCTGCAAATCATGGACTATCT | CAGCTGCAGCGTCATCA | Human |
| *SLC38A7* | CCACCATCTGCTTCGGATTT | ATGTAGACAGCGAGGGCTAT | Human |
